# Genome-Wide Identification and Characterization of Basic Pentacysteine Transcription Factors in *Brassica napus*

**DOI:** 10.3390/plants14071136

**Published:** 2025-04-06

**Authors:** Huan Hu, Yuqin Jiang, Chiyuan Liu, Ying Zhang, Mingxun Chen, Zijin Liu

**Affiliations:** 1 Shaanxi Key Laboratory of Crop Heterosis, College of Agronomy, Northwest A&F University, Yangling 712100, China; huhuan@nwafu.edu.cn (H.H.); jyq32322021@nwafu.edu.cn (Y.J.); liuchiyuan@nwafu.edu.cn (C.L.); 2 Department of Ecological and Environmental Engineering, Yangling Vocational & Technical College, Yangling 712100, China; yingzhang28@126.com

**Keywords:** gene family, *Brassica napus*, BPC, genome-wide identification, expression pattern

## Abstract

BARLEY B-RECOMBINANT/BASIC PENTACYSTEINE (BBR/BPC), a plant-specific transcription factor family, is a group of GAGA_motif binding factors controlling multiple developmental processes of growth and response to abiotic stresses. BPCs recruit histone remodeling factors for transcriptional repression of downstream targets. However, the information about BnaBPCs from *Brassica napus* remains unclear. Here, we identified 25 *BnaBPC* genes that were mainly localized in the nucleus, randomly localized on 16 chromosomes, and grouped into three subfamilies based on phylogenetic analysis. Twenty-five *BnaBPC* genes exhibit syntenic relationships with *AtBPC* genes, and the polypeptides encoded by *BnaBPC* genes within the same subfamily share similar conserved motifs and protein domains. The expansion of *BnaBPC* genes underwent whole-genome duplication events and purifying selection in genomes, and all the *BnaBPC* genes had the same conserved GAGA binding domains. Additionally, the promoter of each *BnaBPC* gene consisted of various *cis*-elements associated with stresses, phytohormones, and growth and development. Notably, the seed-specific regulatory element was found only in the *BnaC04.BPC4* promoter. Further expression pattern analysis showed that *BnaBPC* members are widely expressed in stems, buds, developing seeds and siliques. These findings provide insights into *BnaBPC* genes and enrich our understanding of their functional characterization in *B. napus*.

## 1. Introduction

Oilseed rape (*Brassica napus* L., 2n = 38), cultivated worldwide as the second largest oil crop, serves as a vital source of edible oil [1]. Beyond its primary role as an oilseed, *B. napus* is also cultivated for green manure, vegetable consumption, animal feed, honey production, and ornamental purposes [2], highlighting its diverse economic and ecological importance. Since the demand for vegetable oil has sharply increased in recent years, the major goals for *B. napus* breeding are to improve seed productivity and seed oil content. However, global warming and climate change have led to a concerning rise in the intensity and frequency of abiotic stresses, which pose significant challenges to crop production worldwide [3,4,5]. *B. napus* is particularly vulnerable to these stresses, impacting its growth, development, and ultimately, seed yield [6,7,8,9]. Therefore, identifying and functionally analyzing key genes associated with both seed productivity and abiotic stress tolerance is crucial for developing climate-resilient oilseed rape cultivars through genetic improvement strategies.

As a plant-specific transcription factor family, BASIC PENTACYSTEINE (BPC) proteins are found throughout land plants. The *Arabidopsis thaliana* genome encodes seven *BPC* genes (*AtBPC1*~*AtBPC7*) that are divided into three classes based on protein domain structures and sequence similarity, namely class I (*AtBPC1*~*AtBPC3*), class II (*AtBPC4*~*AtBPC6*), and class III (*AtBPC7*) [10]. All seven AtBPC proteins consist of a conserved DNA-binding domain at the C-terminus, which can recognize and bind to the GAGA-rich box in the promoters of their target genes [10,11]. AtBPC1 negatively regulates the development of ovule and embryo by directly repressing the expression of *AtINNER NO OUTER*, *AtSEEDSTICK*, and *AtFUSCA3* [11,12,13]. AtBPC2 negatively regulates osmotic stress tolerance in the seedling growth stage by directly repressing the expression of *AtLATE EMBRYOGENSIS ABUNDANT4-5* [14]. The ectopic expression of *AtBPC3* impedes the formation of leaf margin by suppressing the expression of *AtTEOSINTE BRANCHED1*, *AtCYCLOIDEA*, and *AtPROLIFERATING CELL FACTORs* [15]. *AtBPC5* is thought to be a pseudogene that encodes a functionally inactive protein because of the presence of an in-frame stop codon [10,11]. In addition, *AtBPCs* act redundantly to regulate plant growth and development and respond to salt stress. For example, the members of *AtBPC: AtBPC1*, *AtBPC2*, *AtBPC4*, and *AtBPC6* modulate root development by directly inhibiting *AtABSCISIC ACID INSENSITIVE4* expression [16]. The *atbpc1 atbpc2* double mutant displays minor vegetative and reproductive deformities, which are more severe in the *atbpc1 atbpc2 atbpc4 atbpc6* quadruple mutant [10,16]. Class I BPCs collaborate redundantly to control inflorescence and flower development by reducing the expression levels of *AtSHOOTMERISTEMLESS* and *AtBREVIPEDICELLUS/KNAT1* [17]. AtBPC1 together with AtBPC2 promotes β-1,4-galactan accumulation and enhances salt tolerance by repressing *AtGALACTAN SYNTHASE 1* expression [18]. Apart from the redundant roles across AtBPC members, AtBPC3 has been characterized by its antagonistic relationship with other AtBPC proteins. The dwarfism and short primary inflorescence of the *atbpc1 atbpc2 atbpc4 atbpc6* quadruple mutant can be restored by *AtBPC3* mutation [10]. Additionally, AtBPC3 has been shown to antagonize the functions of other AtBPCs in regulating the circadian clock and flowering time, with overexpression of AtBPC3 leading to growth defects similar to those seen in the higher-order *bpc* mutants (*atbpc1 atbpc2 atbpc4 atbpc6*) [15]. These findings indicate that AtBPC3 plays a key role as a repressor in coordinating circadian rhythms and plant development through its antagonistic interactions with other AtBPC family members. The *BPC* transcriptional factor family has also been identified from other plant species, such as *Glycine max* [19], *Hordeum vulgare* [20], *Oryza sativa* [21], and *Cucumis sativus* [22]. Functional analysis has shown that these *BPC*s exhibit important roles in regulating the development of leaves, inflorescence, flowers, ovules, and embryos; the formation of later roots; and the response to salt and osmotic stresses [19,20,21,22]. However, the function of BnaBPC members remains unknown in *B. napus*.

The current study identified 25 BnaBPCs from the *B. napus* genome and executed comprehensive phylogenetic, gene structure, conserved motifs, and gene expression analysis. These findings enhance our understanding of BnaBPCs and provide a foundation for further studies on their biological functions.

## 2. Results

### 2.1. Identification and Characterization of BnaBPC Family Members

**Figure 1:**
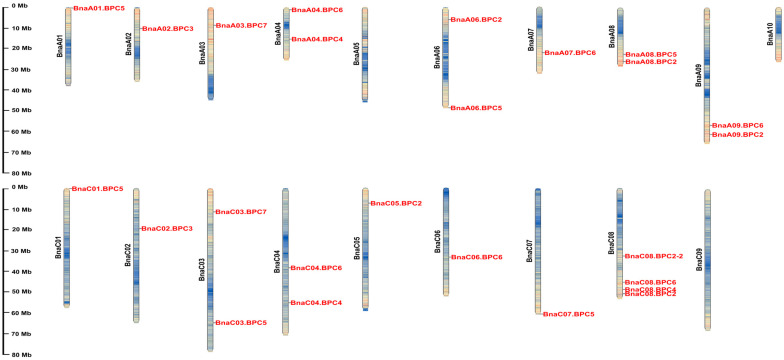
Location of *BnaBPC* genes on the chromosomes of *B. napus* cultivar ‘ZS11’. The chromosome size is expressed by length. The chromosome number is displayed on the left of each chromosome. The color variation represents the density of the region, where blue indicates low, yellow indicates medium, and red indicates high. A black line indicates the relative position of each *BnaBPC* gene. The scale bar on the left side is displayed as megabases (Mb).

To identify BPC family members in the *B. napus* genome, we performed a local BLASTP search against the *B. napus* cultivar ‘Zhong Shuang 11’ (‘ZS11’) genome using the amino acid sequences of AtBPC proteins as query sequences. ‘ZS11’ is a representative accession of the Yangtze River Basin ecotype and has been extensively used in rapeseed genomics research due to its high oil content, superior disease resistance, and completed genome assembly [23]. A total of 25 BnaBPC proteins were predicted from the ‘ZS11’ genome (Figure 1, Supplementary Table S1). These BnaBPC proteins were categorized and named as BnaBPC2, BnaBPC3, BnaBPC4, BnaBPC5, BnaBPC6, and BnaBPC7 based on the sequence similarity with AtBPC proteins. Unfortunately, no homolog of AtBPC1 was identified in the genome of *B. napus* cultivar ‘ZS11’ (Figure 1). In addition, chromosomal analysis showed that all *BnaBPC* genes were distributed on 16 different chromosomes, except for chromosomes A5, A10, and C9. Among the 25 *BnaBPC* genes, 12 were localized in the AA subgenome, including 3 *BnaBPC2* genes, 1 *BnaBPC3* gene, 1 *BnaBPC4* gene, 3 *BnaBPC5* genes, 3 *BnaBPC6* genes, and 1 *BnaBPC7* gene. The remaining 13 *BnaBPC* genes were localized in the CC subgenome, consisting of 3 *BnaBPC2* genes, 1 *BnaBPC3* gene, 2 *BnaBPC4* genes, 3 *BnaBPC5* genes, 3 *BnaBPC6* genes, and 1 *BnaBPC7* gene (Figure 1). These results suggested that *BnaBPC* genes are unevenly distributed across the AA and CC subgenomes, with subgenome-specific expansion in *BnaBPC2*/*5*/*6* paralogs.

The average length of the BnaBPC proteins was 308 amino acids, with the longest being 570 amino acids (BnaA06.BPC2) and the shortest length being 134 amino acids (BnaC08.BPC4) (Table 1). The analysis of the physicochemical properties of the BnaBPC proteins showed that their molecular weights ranged from 14.97 kDa (BnaC08.BPC4) to 65.34 kDa (BnaC05.BPC2) (Table 1). The theoretical isoelectric point (pI) of these proteins ranged from 8.66 to 10.46, with all BnaBPC family members having a pI greater than 7. Proteins were classified as unstable if their instability index exceeded 40, and stable if the index was below 40 [24]. Among the BnaBPC proteins, the instability index ranged from 26.50 to 59.04 (Table 1). Three proteins had an instability index below 40, while 22 proteins had an instability index above 40 (Table 1). Subcellular localization predictions showed that all 25 BnaBPC proteins were localized to the nucleus (Table 1), consistent with their potential role as transcription factors.

### 2.2. Phylogenetic and Genome Synteny Analysis of BnaBPC Proteins

**Figure 2:**
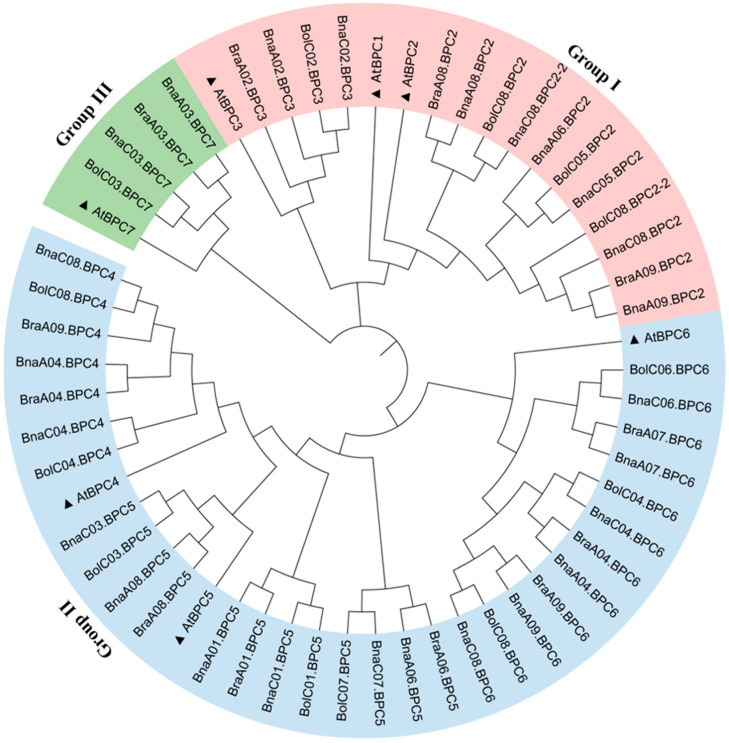
Phylogenetic analysis of BPC proteins in *A. thaliana*, *B. napus*, *B. oleracea*, and *B. rapa*. BPC family proteins are grouped into I, II, and III subfamilies, indicated by red-, blue-, and green-colored arcs, respectively. The black triangle indicates AtBPC proteins. At: *A. thaliana*, Bna: *B. napus*, Bra: *B. rapa*, and Bol: *B. oleracea*.

To investigate the evolutionary relationships of *BPC* gene family members, we constructed a phylogenetic tree using the full-length amino acid sequences of BPC proteins from *B. napus*, *Brassica oleracea*, *Brassica rapa*, and *A. thaliana,* with MUSCLE [25] alignment in MEGA 11.0. The 57 proteins were classified into three subfamilies (Figure 2), recapitulating the *A. thaliana* classification framework [11]. However, the analysis revealed Brassica-specific lineage sorting, notably the absence of *AtBPC1* orthologs in subfamily I (Figure 2). There were 18 individuals in subfamily I, 34 in subfamily II, and 5 in subfamily III (Figure 2). Additionally, all the BnaBPC proteins were closely related to their homologs in *A. thaliana* (Figure 2).

**Figure 3:**
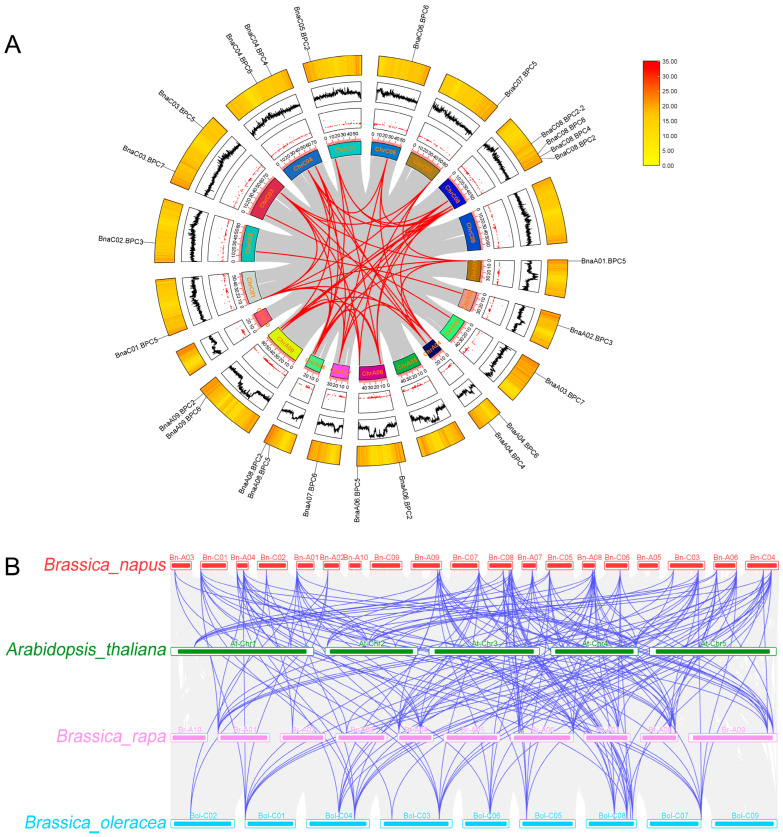
Circular representations of *BnaBPC* chromosomal dispersal and inter-chromosomal interactions, as well as synteny analysis of *BPC* genes in *B. rapa*, *B. oleracea*, *B. napus*, and *A. thaliana.* (**A**) Analysis of syntenic relationships between *BnaBPC* paralog pairs. In the schematic image, red lines show duplicate pairs of *BnaBPCs*. (**B**) Analysis of syntenic relationships between *BraBPCs*, *BolBPCs*, *BnaBPCs*, and *AtBPCs*, as indicated by blue lines.

BLAST and MCScanX were used to identify gene duplication events of *BnaBPCs*, which were visualized using TBtools (Advance Circle) (Figure 3A). Several Whole Genome Duplication (WGD) events were observed, including a pair of tandem duplicates on chromosome ChrC08 (*BnaC08.BPC2::BnaC08.BPC2-2*). Additionally, 50 pairs of homologous genes were identified: 9 paralog pairs came from the genome of group A, 11 came from the genome of C, and 30 came from WGD events in the genomes of groups A and C (Supplementary Table S2).

**Figure 4:**
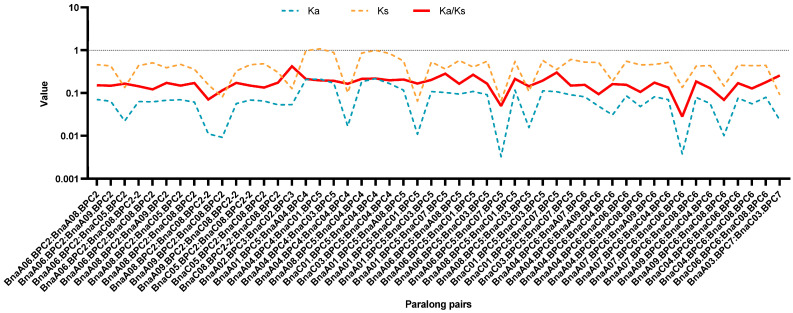
Evolutionary selection analysis of BnaBPC paralog pairs based on Ka/Ks ratios. Blue lines represent Ka values (nonsynonymous substitution rate), orange lines denote Ks values (synonymous substitution rate), and red lines indicate Ka/Ks ratios. Ratios > 1 suggest positive selection, <1 indicate purifying selection, and ≈1 represent neutral evolution. The *X*-axis shows paralog pairs, while the *Y*-axis represents the corresponding values of Ka, Ks, and Ka/Ks ratios.

A collinearity analysis of *A. thaliana*, *B. napus*, *B. oleracea*, and *B. rapa* was conducted to explore the evolutionary mechanisms of the *BPC* family members in different species. The results revealed that 30 paralog pairs were formed between *B. napus* and *A. thaliana*, 72 pairs between *B. napus* and *B. rapa*, and 73 pairs between *B. napus* and *B. oleracea* (Figure 3B, Supplementary Table S3). These results suggested that segmental duplication is the primary mechanism driving the evolution of BPC members. To assess the evolutionary mode of *BnaBPCs*, the Ka/Ks ratios for 50 homologous gene pairs were calculated. The Ka/Ks ratios of all pairs were found to be less than 1 (Figure 4, Supplementary Table S4), indicating that the evolution of BnaBPCs is primarily governed by purifying selection.

### 2.3. Gene Structures and Amino Acid-Conserved Structures of BnaBPC Genes

**Figure 5:**
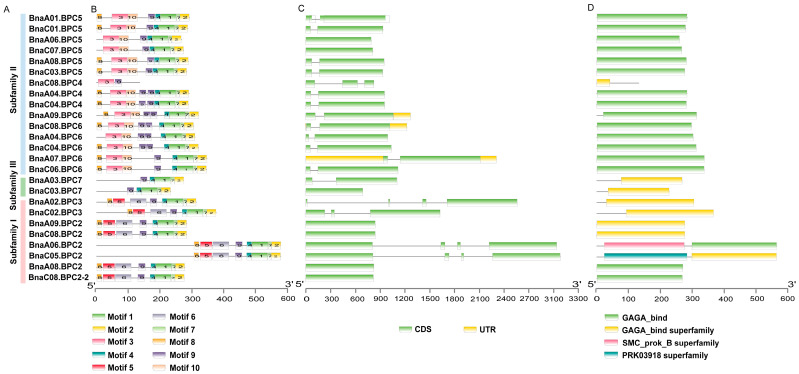
Schematic diagram of gene structures, conserved motifs, and conserved protein domains of *BnaBPC* genes. (**A**) Subfamily classification. (**B**) The conserved motifs of BnaBPC proteins. The motifs, numbers 1–10, are represented with different colored boxes in the BnaBPC proteins. The scale at the bottom is used to estimate the length of proteins. (**C**) Gene structures of *BnaBPC* genes. Green boxes represent exons, and light gray lines indicate introns. Yellow boxes indicate the untranslated regions (UTRs). The scale at the bottom represents the length of genes. (**D**) The structural domains of BnaBPC proteins. The scale at the bottom is used to estimate the length of proteins.

To further gain a deeper understanding of their characteristics, we analyzed the gene structure, conserved motifs, and domains of *BnaBPC* genes using MEME, TBtools, and the Conserved Domain Database (CDD). The analysis results revealed that the highly conserved motifs (motifs 1, 2, 4, 7, 9) were present in all BPC proteins except for BnaC08.BPC4 (Figure 5B). BnaBPC proteins within the same subfamily shared similar motif compositions, while proteins from different subfamilies exhibited significant variations in their motif compositions (Figure 5B). Specifically, proteins in subfamilies I and II contained motifs 1, 2, 3, 4, 7, 9, and 10, except for *BnaC08.BPC4*, which only includes motifs 3 and 9 (Figure 5B). Additionally, with the exception of *BnaC08.BPC4*, *BnaA06.BPC5*, *BnaC07.BPC5*, and *BnaA04.BPC6*, all proteins in subfamilies I and II contained motif 8. Subfamily III proteins only contained motifs 1, 2, 4, 7, and 9 (Figure 5B). Gene structure analysis revealed that only *BnaA07.BPC6*, *BnaA09.BPC6*, and *BnaC08.BPC6* contained the untranslated regions, while other *BnaBPC* genes did not (Figure 5C). Furthermore, *BnaBPC* genes typically contained one to three introns, with the exception of *BnaA06.BPC5*, *BnaC07.BPC5*, *BnaC03.BPC7*, *BnaA09.BPC2*, *BnaC08.BPC2*, *BnaA08.BPC2*, and *BnaC08.BPC2-2* (Figure 5C).

Members of the BPC family consist of two main structural domains: the GAGA-binding domain and the GAGA-binding superfamily domain. Subfamilies II contains one GAGA-binding domain, except for BnaC08.BPC4, which has one GAGA-binding superfamily. In contrast, most members of subfamily I and III possess one GAGA-binding superfamily domain. Notably, BnaA06.BPC2 and BnaC05.BPC2 have the SMC_prok_B_superfamily and PRK03918 superfamily domains, respectively (Figure 5D).

In summary, the similar gene structures of the *BnaBPC* genes, along with the conserved motifs and protein domains in their encoded polypeptides within the same subfamily, strongly support the results of the subfamily classification obtained from the phylogenetic analysis.

### 2.4. Cis-Element Analysis of BnaBPC Gene Promoters

**Figure 6:**
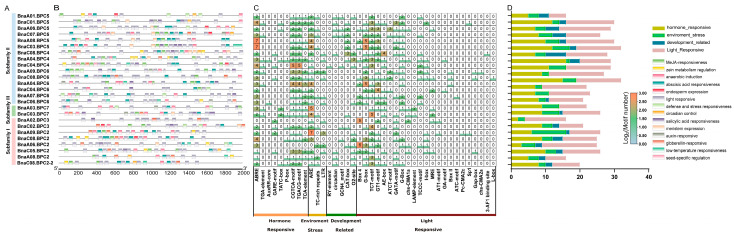
Predicted *cis*-elements in the promoter of *BnaBPC* genes. (**A**) Subfamily classification. (**B**) The location of different *cis*-elements on the 2000 bp promoter sequence upstream of the start codon of each *BnaBPC* gene. The different colored boxes indicate different *cis*-elements. (**C**) The numbers of the different *cis*-elements. The numbers and color scale represent the Log_2_ (motif number). (**D**) Analysis of the distribution and classification of *cis*-elements in *BnaBPCs*.

To reveal the potential roles and regulatory mechanisms of *BnaBPC* genes, we selected the 2000 bp promoter sequence upstream of the start codon of each *BnaBPC* gene to predict *cis*-elements. A total of 357 *cis*-elements were predicted and mainly associated with hormone-responsive, development-related, environment stress, and light-responsive in the *BnaBPC* promoters (Figure 6; Supplementary Table S5). Among these *cis*-elements, the light-responsive element was particularly prominent (Figure 6B,C), indicating that *BnaBPC* genes may be regulated by light and subsequently participate in plant growth and development. In addition, the promoters of *BnaBPC* genes contained several environment-stress-responsive elements. Particularly, 24 *BnaBPC* genes possessed anaerobic induction elements, 16 *BnaBPC* genes included defense and stress-responsive elements, and 10 *BnaBPC* genes carried low-temperature responsive elements (Figure 6B). The *cis*-elements related to MeJA responsiveness and abscisic acid responsiveness were more prevalent than those related to salicylic acid responsiveness, gibberellin responsiveness, and auxin responsiveness (Figure 6B). These findings indicate that *BnaBPC* genes may play a role in regulating plant growth and development by responding to various hormonal signals. Furthermore, the development-related elements, such as meristem expression, zein metabolism regulation, circadian control, endosperm expression, and seed-specific regulation, were also found in the promoters of some *BnaBPC* genes (Figure 6B). Notably, the seed-specific regulation element was only presented in the *BnaC04.BPC4* promoter (Figure 6), suggesting *BnaC04.BPC4* may function in seed development in a tissue-specific manner.

In conclusion, *BnaBPC* genes could play vital roles in ensuring plant normal growth and development under different growing statuses and environmental conditions independently or synergistically.

### 2.5. Expression Profiles of BnaBPC Genes in Various Tissues

**Figure 7:**
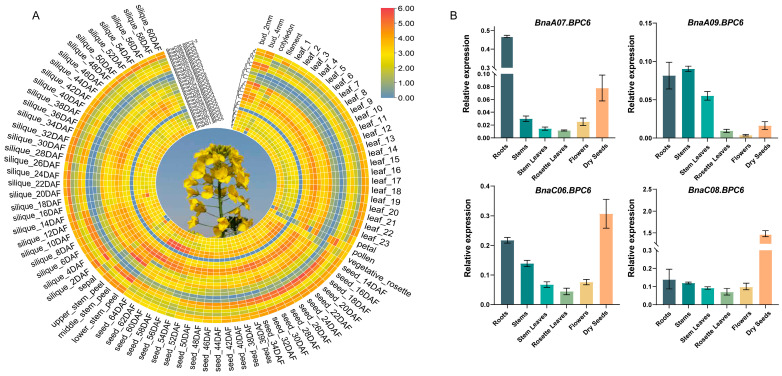
Expression profiles of *BnaBPC* genes in various tissues of *B. napus* cultivar ‘ZS11’. (**A**) Publicly available RNA-Seq data of different tissues, as indicated at the bottom, were obtained from the BnIR database. The color scale represents relative expression levels, with low expression in blue and high expression in red. The scale bar represents Log_2_(TPM)-normalized expression. The axes indicate *BnaBPCs* and the phylogenetic tree. (**B**) RT-qPCR validation of *BnaA07.BPC6*, *BnaA09.BPC6*, *BnaC06.BPC6*, and *BnaC08.BPC6* in different tissues, with *BnaGAPDH* serving as the internal control. The error bars represent the standard deviation (SD) of three biological replicates.

To further investigate the potential functions of *BnaBPCs*, their expression patterns were analyzed in the stems, cotyledons, rosette leaves, buds, filaments, petals, pollens, sepals, developing siliques, and developing seeds using publicly available transcriptome data of *B. napus* cultivar ‘ZS11’ (Supplementary Table S6). The results showed that six *BnaBPC2* genes were strongly transcribed in the stems, pollens, and early stages of seed development (Figure 7A), indicating that *BnaBPC2* genes may play a conserved role in controlling flower and seed development. The expression levels of *BnaA02.BPC3* and *BnaC02.BPC3* were higher in the buds and pollens, respectively, while being undetectable in other tissues (Figure 7A), implying that *BnaBPC3* genes regulate flower development in a tissue-specific manner. Almost all *BnaBPC4* genes were expressed across various tissues. In contrast, *BnaC08.BPC4* showed no detectable expression (transcriptome data of zero) in any of the analyzed tissues (Figure 7A). The six *BnaBPC5* genes were expressed in the stems, rosette leaves, and buds, except for *BnaC01.BPC5*, which remained at a relatively higher transcription level in the filaments, petals, and sepals (Figure 7A). Moreover, *BnaA06.BPC5* and *BnaC07.BPC5* were highly expressed in the developing seeds at 16 days after pollination (DAP), whereas other *BnaBPC5s* increased progressively afterward at the late stages of seed maturation and reached the maximal level at 60~64 DAP (Figure 7A). Furthermore, most of the *BnaBPC6* genes exhibited a higher transcriptional level in stems and leaves, and *BnaBPC7* genes had relatively higher transcription levels in the buds and developing seeds at 56~64 DAP (Figure 7A).

To validate the BnIR expression database, total RNA was extracted from different tissues of the *B. napus* cultivar ‘ZS11’, including the roots, stems, stem leaves, rosette leaves, flowers, and dry seeds. Quantitative analysis revealed distinct expression patterns of *BnaBPC6* paralogs across tissues: *BnaA07.BPC6* and *BnaC06.BPC6* were predominantly expressed in the roots and dry seeds; *BnaA09.BPC6* was primarily localized in the roots, stems, and stem leaves; and *BnaC08.BPC6* showed dominant expression in dry seeds (Figure 7B). Further comparative analysis identified tissue-specific expression hierarchies: in the root tissues, *BnaA07.BPC6* exhibited the highest expression level among the four paralogs, followed by *BnaC06.BPC6*; in dry seeds, *BnaC08.BPC6* displayed the most abundant transcripts, with *BnaC06.BPC6* ranking second. These tissue-specific expression patterns, quantitatively validated by RT-qPCR, were fully consistent with the transcriptome sequencing data, reinforcing the functional relevance of *BnaBPC6* paralogs in the regulating growth and developmental processes of *B. napus*.

Our comprehensive analysis indicates that BnaBPC family members function as master regulators governing multiple biological pathways, exhibiting both functional redundancy and specialization across different tissue types.

## 3. Discussion

Plant growth and development are controlled by multiple and precise regulatory networks that coordinate various external environmental and endogenous signals under different growing statuses and environmental conditions [26]. Among them, transcription regulation is crucial for balancing multi-level signals [27]. Gene regulation goes through a series of complex processes in which transcription factors play an important role. Transcription factors exhibit a wide range of functions in plant growth, including plant development and the formation of the overall morphological diversity of plants. Herein, gaining insight into the structure and function of transcription factors is essential to unravel the regulatory mechanisms that govern plant development and growth.

BPC is a plant-specific transcription factor family, and increasing evidence has revealed that *BPC* genes from *A. thaliana* and other crops are essential for controlling growth and development, as well as for response to biotic and abiotic stresses [10,17]. However, there is no relevant report on the type, quantity, structure, and function of the BPC family in *B. napus*. In this study, we utilized the *AtBPC* genes as a reference to investigate the *BnaBPC* gene family members throughout the entire genome. Additionally, we analyzed the gene structure and the gene evolutionary relationship of the *BPC* genes in different species. The expression patterns of the *BnaBPC* genes under different tissues were explored. This will provide useful data for high yield and stress tolerance breeding of *B. napus*.

*B. napus* is an allotetraploid crop formed by natural hybridization between two diploid progenitors, *B. rapa* and *B. oleracea*. Its evolution is accompanied by chromosomal duplication, rearrangement, and deletion, resulting in an average of two to eight paralogs of each *A. thaliana* locus in the *B. napus* genome [28,29,30]. Accordingly, two to six paralogs are homologous to each *AtBPC* in the genome of *B. napus* (Figure 1). We successfully identified 25 *BnaBPC* genes and divided them into three subfamilies (Figure 1 and Figure 2), which was consistent with the previous study, which showed that the *BPC* members encoded by the *A. thaliana* genome fall into three subfamilies [10]. The 25 *BnaBPCs* were unevenly distributed on 16 chromosomes and had different genetic structural features in each group. It is worth mentioning that there were no homologs of *AtBPC1* identified in the *B. napus* genome (Figure 1), which might be due to the deletion occurrence during the evolution process. The subfamilies II and III shared similar members between *A. thaliana* and *B. napus*, while *AtBPC1* homologs were missing from subfamily I (Figure 2), further demonstrating that the homologs of *AtBPC1* disappeared during the evolution process.

An analysis of the syntenic relationships between species indicated that the *BPC* genes of *B. napus* were mainly derived from genome evolution with *B. oleracea* and *B. rapa*. The evolution mode was purifying selection, which led to the formation of tandem duplicates on chromosome ChrC08 during the WGD process (Figure 3 and Figure 4). Predicted subcellular localization suggests that BnaBPCs are localized in the nucleus, implying their potential role as transcription factors. This observation indicated that the overall functions of the gene family have been conserved, although the functions of genes in each subgroup were different. Moreover, *BnaBPC* members within the same subfamily possessed similar exon/intron structures and conserved motifs, suggesting a closer evolutionary relationship among the same subfamily members (Figure 5). There were significant differences in gene structure and sequence lengths of *BnaBPC* members in different subgroups (Figure 5, Table 1), indicating functional diversification among the members of distinct subfamilies. It has been reported that introns can improve the content of mRNA by affecting transcription and can also enhance mRNA translation efficiency [31]. The *BnaBPC* genes may have different biological activities due to their different intron structures. Further analysis of conserved domains and motifs showed the presence of typical motifs in all BnaBPCs (Figure 5). High sequence similarity among motif sequences within each subgroup (Figure 5) indicated that BPC members of each subgroup potentially share similar functions. Additionally, all of the BnaBPC proteins contained a conserved GAGA-binding domain or a GAGA-binding superfamily (Figure 5) that specifically interacts with GA-rich box *cis*-elements [11]. Previous studies showed that AtBPC6 interacts with LHP1 and then recruits PRC1 and PRC2 members to the GAGA structural domains of polycomb-responsive DNA elements of the target genes [10]. Given the conservation of the GAGA-binding domain in BnaBPC proteins, we hypothesize that BnaBPC proteins may similarly recruit PRC1 and PRC2 to target genes via GAGA motifs, acting similarly to their *A. thaliana* counterparts. Notably, previous studies have shown that the phylogenetic relationships among AtBPC proteins do not precisely mirror the functional relationship, and *AtBPC* genes function redundantly and antagonistically to regulate plant growth and development [10,16]. Therefore, the detailed roles and their specific functions of BnaBPC proteins need further investigation.

Transcription regulation is indispensable for plant growth and development [32]. The *cis*-elements in the promoter are essential for the expression and functions of genes [33,34]. Our results showed that the promoters of *BnaBPC* genes contained various *cis*-elements that are associated with development-related, hormone-responsive, light-responsive, and environment stress responses (Figure 6), including ABRE-motif, ARE-motif, and Box 4-motif, etc. Together with the results of the different expression levels of the *BnaBPC* genes under different tissues (Figure 7A), these findings suggest that *BnaBPC* genes might play a crucial role in plant growth, development, and the environmental stress responsiveness of plants through a variety of hormone-regulatory pathways and physiological processes. Moreover, six *BnaBPC2* genes were strongly transcribed in the stems, pollen, and early stages of seed development (Figure 7A), suggesting that *BnaBPC2* genes may play an important role in the growth of stems and pollen and the early development of seeds in *B. napus*. Previous studies showed that AtBPC6 can bind the GAGA_motif, resulting in the recruitment of PRC1 and PRC2 components to Polycomb-responsive DNA element-like GAGA motifs, which play key roles in development by repressing large numbers of genes involved in various functions [35]. Our studies showed that all six BnaBPC6s contained one GAGA-binding sequence and various *cis*-elements that are associated with hormone responsiveness and light responsiveness (Figure 6), suggesting that *BnaBPC6* genes may play a role in growth and development by the same mechanism as *AtBPCs*. The differential expression patterns of *BnaBPC6* paralogs across various tissues (Figure 7B), as revealed by RT-qPCR analysis, strongly suggest their specialized roles in growth and development regulation. The distinct expression profiles of the four *BnaBPC6* copies indicate potential functional diversification in different tissues, with each paralog potentially serving as a major regulatory factor in specific developmental contexts. The presence of hormone-responsive *cis*-elements in *BnaBPC6* promoters further supports their potential role in coordinating developmental transitions through hormonal signaling pathways, consistent with the known function of *AtBPC6* in *A. thaliana*. However, the specific biological functions of these paralogs may require further validation using genetic materials, such as targeted mutants or transgenic plants, to establish direct causal relationships between their expression patterns and developmental phenotypes.

In conclusion, 25 *BnaBPC* genes were identified from the *B. napus* cultivar ‘ZS11’ genome and divided into three subfamilies. They shared high similarity in gene structure, conserved motif composition, and conserved protein domains within the same subfamily. Moreover, all of the *BnaBPC* genes contained various *cis*-elements and were broadly expressed in various tissues. These results provide a basis for the further functional analysis of *BnaBPC* genes in *B. napus* and other plant species.

## 4. Materials and Methods

### 4.1. Identification of BnaBPCs in the B. napus Genome

The amino acid sequences of the BPC family members in *A. thaliana* were obtained from the TAIR database (https://www.arabidopsis.org/, accessed on 5 September 2024). The reference genome of *B. napus* cultivar ‘ZS11’ was downloaded from the *B. napus* pan-genome information resource (BnPIR) database (http://cbi.hzau.edu.cn/bnapus/index.php, accessed on 5 September 2024). Using AtBPC amino acid sequences as queries, we performed Hidden Markov Model (HMM) and local BLASTP searches against the *B. napus* cultivar ‘ZS11’ genome to identify BnaBPC members, employing default E-value settings. All candidate sequences of BnaBPCs were confirmed using the Pfam (http://pfam.xfam.org, accessed on 6 September 2024) [36] and SMART (http://smart.embl-heidelberg.de/, accessed on 6 September 2024) databases [37]. The BnaBPC members were named based on their order of arrangement on the chromosomes of the *B. napus* genome.

### 4.2. Phylogenetic Analysis and Classification of the BnaBPC Family

The multiple sequence alignment of AtBPC and BnaBPC amino acid sequences was performed using ClustalW with default parameters [38]. The phylogenetic tree was constructed using the neighbor-joining (NJ) method with the *p*-distance model in MEGA 11.0 software, and bootstrap values were calculated based on 1000 replicates [38,39]. The AtBPC family members were used as a reference to classify the *BnaBPC* family members.

### 4.3. Chromosomal Localization of BnaBPCs

The chromosomal positions of *BnaBPCs* were queried from the *B. napus* genome annotation information and visualized using the TBtools software (v2.210) [40]. Intragenomic syntenic relationships among BnaBPCs were identified through BLAST+ (v2.13.0) pairwise alignments, followed by collinear block analysis with MCScanX software (v1.1) using default parameters [41].

### 4.4. Analysis of Gene Structures, Conserved Motifs, and Conserved Protein Domains of BnaBPCs

The gene structures of *BnaBPCs* were displayed based on *B. napus* genome annotation information by the TBtools software (v2.210) [40]. The conserved motifs of BnaBPC proteins were identified using the Multiple Expectation Maximization for Motif Elicitation (MEME) online program (https://meme-suite.org/meme/, accessed on 10 September 2024) [42] with the maximum number of motifs and the optimum width of each motif falls between 10 and 100 residues. The conserved domains of BnaBPC proteins were predicted using the Conserved Domains Database (CDD) (https://www.ncbi.nlm.nih.gov/Structure/cdd/cdd.shtml, accessed on 10 September 2024).

### 4.5. Cis-Element Analysis in the Promoters of BnaBPCs

The 2000 bp promoter sequence upstream of the start codon of each *BnaBPC* gene was selected from the reference genome of *B. napus* cultivar ‘ZS11’ and then submitted to the PlantCARE database (https://bioinformatics.psb.ugent.be/webtools/plantcare/html/, accessed on 14 September 2024) for *cis*-element prediction. The predicted *cis*-elements were visualized by the TBtools software (v2.210).

### 4.6. Expression Patterns of BnaBPCs

Transcriptome data from different tissues of the *B. napus* cultivar ‘ZS11’ were downloaded from the BnPIR database (http://cbi.hzau.edu.cn/bnapus/index.php, accessed on 20 September 2024). Expression standardization of *BnaBPC* genes was constructed using the DSEeq2 R package, and the heatmaps were performed using the TBtools software. To verify the gene expression data of BnIR, the SteadyPure Plant RNA Extraction Kit (Accurate Biology, Changsha, China) was utilized to extract RNA from the roots, stems, stem leaves, rosette leaves, flowers, and dry seeds of ‘ZS11’. An EasyScript One-Step gDNA Removal and cDNA Synthesis SuperMix (TransGen, Beijing, China) was used to obtain cDNA from 1 µg RNA. Specific primers were designed using Primer-BLAST (https://www.ncbi.nlm.nih.gov/tools/primer-blast, accessed on 20 September 2024) (Supplementary Table S7). RT-qPCR was performed with three biological replicates using SYBR Green Master Mix (Cofitt, Hong Kong, China), and each biological replicate had three technical replicates.

## Figures and Tables

**Table 1:** Physical and chemical properties of BPC family proteins in *B. napus*.

Gene ID	Gene Name	Location	Length	Molecular Weight (Da)	pI	Instability Index	Aliphatic Index	GRAVY	Subcellular Localization
BnaA06G0096700ZS	*BnaA06.BPC2*	A06:5788186..5791223	570	65,234.18	8.66	55.78	68.6	−0.791	nuclear
BnaA08G0270900ZS	*BnaA08.BPC2*	A08:26026857..26027678	273	30,490.56	9.51	57.73	52.53	−0.84	nuclear
BnaA09G0631800ZS	*BnaA09.BPC2*	A09:61219562..61220401	279	31,216.44	9.65	53.63	50.36	−0.853	nuclear
BnaC05G0118800ZS	*BnaC05.BPC2*	C05:7417299..7420376	569	65,338.51	8.92	57.30	71.97	−0.764	nuclear
BnaC08G0230300ZS	*BnaC08.BPC2-2*	C08:32814996..32815811	271	30,205.25	9.51	54.96	51.48	−0.845	nuclear
BnaC08G0489500ZS	*BnaC08.BPC2*	C08:50925950..50926789	279	31,200.44	9.65	52.14	51.04	−0.837	nuclear
BnaA02G0171100ZS	*BnaA02.BPC3*	A02:10179794..10182350	308	34,783.84	9.58	49.55	63.64	−0.605	nuclear
BnaC02G0219700ZS	*BnaC02.BPC3*	C02:19442545..19474170	370	41,534.44	9.55	52.48	65.59	−0.561	nuclear
BnaA04G0137800ZS	*BnaA04.BPC4*	A04:15262407..15263357	286	31,885.16	9.17	34.87	70.70	−0.699	nuclear
BnaC04G0428300ZS	*BnaC04.BPC4*	C04:55284453..55285403	284	31,618.83	9.05	37.51	66.73	−0.708	nuclear
BnaC08G0454400ZS	*BnaC08.BPC4*	C08:48985111..48985935	134	14,968.89	10.46	26.50	82.31	−0.753	nuclear
BnaA01G0002800ZS	*BnaA01.BPC5*	A01:296800..297762	287	32,212.38	9.44	55.38	65.68	−0.819	nuclear
BnaA06G0450000ZS	*BnaA06.BPC5*	A06:48524214..48525002	262	29,681.82	9.27	57.63	66.37	−0.833	nuclear
BnaA08G0198900ZS	*BnaA08.BPC5*	A08:22511939..22512885	284	31,838.19	9.29	54.63	63.63	−0.866	nuclear
BnaC01G0002300ZS	*BnaC01.BPC5*	C01:161333..162266	282	31,760.9	9.46	52.04	66.13	−0.828	nuclear
BnaC03G0671500ZS	*BnaC03.BPC5*	C03:64845945..64846875	279	31,413.76	9.40	55.93	62.33	−0.861	nuclear
BnaC07G0547100ZS	*BnaC07.BPC5*	C07:60724181..60724990	269	30,443.6	9.26	58.41	64.65	−0.863	nuclear
BnaA04G0017300ZS	*BnaA04.BPC6*	A04:1121999..1122989	305	34,288.76	9.35	59.04	58.00	−0.922	nuclear
BnaA07G0211100ZS	*BnaA07.BPC6*	A07:22160943..22163249	341	38,028.13	9.28	52.71	57.01	−0.859	nuclear
BnaA09G0548200ZS	*BnaA09.BPC6*	A09:56780945..56782210	316	35,778.63	9.47	50.79	58.45	−0.876	nuclear
BnaC04G0278000ZS	*BnaC04.BPC6*	C04:38436311..38437344	315	35,646.12	9.25	51.23	55.52	−0.979	nuclear
BnaC06G0222600ZS	*BnaC06.BPC6*	C06:33449718..33450832	340	38,057.17	9.20	52.30	57.44	−0.869	nuclear
BnaC08G0394500ZS	*BnaC08.BPC6*	C08:45482887..45484108	300	33,903.27	9.51	57.66	53.77	−1.010	nuclear
BnaA03G0169700ZS	*BnaA03.BPC7*	A03:8638747..8639847	270	29,757.7	9.92	52.33	72.93	−0.375	nuclear
BnaC03G0196600ZS	*BnaC03.BPC7*	C03:11259402..11260091	229	25,501.36	9.75	58.04	60.00	−0.601	nuclear

## Data Availability

The data presented in this study are available in the article and the Supplementary Materials. For further inquiries, you can contact the corresponding author directly.

## Supplementary Materials

The following supporting information can be downloaded at: https://www.mdpi.com/article/10.3390/plants14071136/s1. Table S1. *BPCs* in *B. napus* and their protein sequences. Table S2. The gene pairs of *BnaBPCs*. Table S3. The gene pairs of *BPCs* in *B. napus*, *B. rapa*, *B. oleracea*, and *A. thaliana*. Table S4. Ka/Ks analysis for paralogous gene pairs of *BnaBPCs*. Table S5. List of predicted *cis*-elements in the promoter of *BnaBPC* genes. Table S6. Expression patterns of *BnaBPCs* from the BnIR. Table S7. List of primers used for RT-qPCR expression analysis.
